# Muscle MCT4 Content Is Correlated with the Lactate Removal Ability during Recovery Following All-Out Supramaximal Exercise in Highly-Trained Rowers

**DOI:** 10.3389/fphys.2016.00223

**Published:** 2016-06-10

**Authors:** Hugo Maciejewski, Muriel Bourdin, Léonard Féasson, Hervé Dubouchaud, Christian Denis, Hubert Freund, Laurent A. Messonnier

**Affiliations:** ^1^Inter-University Laboratory of Human Movement Biology, University of Savoy Mont BlancLe Bourget-du-Lac, France; ^2^French Rowing FederationNogent-sur-Marne, France; ^3^IFSTTAR, LBMC UMR_T9406, Claude Bernard University Lyon 1Oullins, France; ^4^Myology Unit, Neuromuscular Rare Diseases Referent Center of Rhone-AlpsCHU Saint-Etienne, France; ^5^Inter-University Laboratory of Human Movement Biology, University of LyonSaint-Etienne, France; ^6^Laboratory of Fundamental and Applied Bioenergetics, Grenoble Alpes UniversityGrenoble, France; ^7^Institut National de la Santé et de la Recherche Médicale, U1055Grenoble, France

**Keywords:** lactate kinetics, monocarboxylate transporters, rowing, humans, endurance training

## Abstract

The purpose of this study was to test if the lactate exchange (γ_1_) and removal (γ_2_) abilities during recovery following short all-out supramaximal exercise correlate with the muscle content of MCT1 and MCT4, the two isoforms of the monocarboxylate transporters family involved in lactate and H^+^ co-transport in skeletal muscle. Eighteen lightweight rowers completed a 3-min all-out exercise on rowing ergometer. Blood lactate samples were collected during the subsequent passive recovery to assess an individual blood lactate curve (IBLC). IBLC were fitted to the bi-exponential time function: La(t) = [La](0) + A_1_(1 − ${\text{e}}^{-\gamma_{1}^{\text{t}}}$) + A_2_(1 − ${\text{e}}^{-\gamma_{2}^{\text{t}}}$) where [La](0) is the blood lactate concentration at exercise completion and the velocity constants γ_1_ and γ_2_ denote the lactate exchange and removal abilities, respectively. An application of the bi-compartmental model of lactate distribution space allowed estimation of the lactate removal rate at exercise completion [LRR(0)]. Biopsy of the right *vastus lateralis* was taken at rest to measure muscle MCT1 and MCT4 content. Fiber type distribution, activity of key enzymes and capillary density (CD) were also assessed. γ_1_ was correlated with [La](0) (*r* = −0.54, *P* < 0.05) but not with MCT1, MCT4 or CD. γ_2_ and LRR(0) were correlated with MCT4 (*r* = 0.63, *P* < 0.01 and *r* = 0.73, *P* < 0.001, respectively) but not with MCT1 or cytochrome c oxidase activity. These findings suggest that the lactate exchange ability is highly dependent on the milieu so that the importance of the muscle MCT1 and MCT4 content in γ_1_ was hidden in the present study. Our results also suggest that during recovery following all-out supramaximal exercise in well-trained rowers, MCT4 might play a significant role in the distribution and delivery of lactate for its subsequent removal.

## Introduction

During recovery following short high-intensity exercises, the blood lactate curve displays a bi-phasic pattern: the concentrations increase during the first minutes of recovery, reach a peak value (between the first to the ninth minute of recovery depending on duration and intensity of exercise as well as on the training status of the subject), and finally decrease (first rapidly then slowly) to return to near resting levels after 60–90 min of passive recovery (Freund and Gendry, 1978; Messonnier et al., 1997, 2001). The increase of blood lactate concentrations during the first minutes of recovery is explained by the fact that lactate appearance transiently exceeds its removal. The elevated appearance at the end of exercise is mainly related to the release of lactate by the previously active muscles (Juel et al., 1990; Bangsbo et al., 1991). Indeed, there exists an important muscle-to-blood lactate gradient at the end of intense exercise. However, the net lactate release rate from the previously active muscles decreases rapidly to become almost nil after a few minutes into recovery (Juel et al., 1990; Bangsbo et al., 1991), so that the lactate removal rate surpasses rapidly the appearance rate and consequently blood lactate concentrations decrease progressively.

This particular pattern of blood lactate concentrations during recovery can be particularly well-described by a bi-exponential time function (Equation 1) referring to a bi-compartmental model of lactate distribution space (Freund and Zouloumian, 1981a,b; Zouloumian and Freund, 1981a,b):

(1) $$\begin{array}{l} \text{La}\left(\text{t}\right)=\left[\text{La}\right]\left(0\right)+{\text{A}}_{1}\left(1-{\text{e}}^{-\gamma_{1}\text{t}}\right)+{\text{A}}_{2}\left(1-{\text{e}}^{-\gamma_{2}\text{t}}\right) \end{array}$$

The first exponential term (with a high velocity constant: γ_1_) refers to the appearance of lactate in blood during recovery and γ_1_ denotes the lactate exchange ability between the previously active muscle and the blood (Freund et al., 1986). The second exponential term (with a low velocity constant: γ_2_) refers to the disappearance of lactate from the blood and γ_2_ provides information on the lactate removal ability during recovery (Freund et al., 1986).

During recovery, the release of lactate from the previously active muscles and its uptake by consumer tissues for recycling essentially by oxidation, gluconeogenesis and glycogen repletion [(Brooks et al., 1973; Gaesser and Brooks, 1980; Fairchild et al., 2003), see also the “lactate shuttle” concept proposed by (Brooks, 2009)] are mainly mediated by the lactate-H^+^ co-transport via the membrane-bound monocarboxylate transporters (MCTs) (Juel, 2008). Several isoforms have been identified, but in humans skeletal muscle, only MCT1 and MCT4 have been well-described so far (Bonen et al., 2006). MCT1 has been found predominantly in oxidative muscle cells (i.e., type I fibers), whereas MCT4 was found with a large variability in the different fiber types (Pilegaard et al., 1999; Dubouchaud et al., 2000). Their different affinity constant (*K*_*m*_) for lactate (4–6 mM for MCT1 and 28–34 mM for MCT4) has suggested that MCT1 may facilitate influx of lactate into oxidative fibers whereas MCT4 would be involved in efflux of lactate out of glycolytic fibers (Dimmer et al., 2000; Manning Fox et al., 2000). However, lactate and H^+^ flux via MCT1 and MCT4 is driven by the concentration gradient of these ions, meaning that MCT1 and MCT4 can potentially transport lactate and H^+^ in both directions i.e., inwards or outwards (Juel, 1997).

In humans, previous studies have reported correlation between the muscle MCT1 content and the net lactate release rate during exercise (Dubouchaud et al., 2000) or post exercise blood lactate concentrations (Bonen et al., 1998; Messonnier et al., 2007). These results suggested that MCT1 could facilitate lactate release from myocytes during heavy exercise i.e., when the gradients of concentrations are favorable for the efflux of lactate from the muscle cells (Bonen et al., 1998; Messonnier et al., 2007). Rather surprisingly, while *in vitro* (*vide supra*) or animal studies (Bonen et al., 2000) would argue in favor of a role of MCT4 in the efflux of lactate from the myocytes, no correlation, to our knowledge, was reported in the literature between muscle MCT4 content and the net lactate release rate in humans. The net lactate release rate depends on the muscle-to-blood lactate gradient but also on the efficiency of the exchanges, appraised *in vivo* by γ_1_ (Freund et al., 1986; Juel, 1997; Messonnier, 2008). To date, no study has investigated the relationship between the muscle MCT1 and MCT4 content and the lactate exchange ability (γ_1_) at the onset of recovery.

Previously, it has been shown that the velocity constant γ_2_ was significantly correlated with the citrate synthase activity, mitochondrial respiration (Thomas et al., 2004) and MCT1 content of the previously active muscles (Thomas et al., 2005). These results reinforced the idea that oxidation constitutes an important pathway of lactate disappearance during recovery (Brooks et al., 1973; Brooks, 2009) and suggested that MCT1 may be involved in lactate uptake (Juel and Halestrap, 1999) by the previously active muscles. However, no studies have investigated the relationship between muscle MCTs content and γ_2_ in a homogeneous group of endurance-trained athletes so far. Furthermore, if the roles of MCT1 and MCT4 have been admittedly investigated after short high-intensity exercises (Thomas et al., 2004, 2005), their roles after exercises leading to extreme lactate accumulations remain unknown.

The aim of the present study was thus to explore the possible links between the muscle MCT1 and MCT4 contents and γ_1_ and γ_2_ determined during recovery following extremely hard exercises (involving maximally the nonoxidative glycolytic energy pathway) performed by long-term endurance-trained athletes. More specifically, our hypotheses are (i) that muscle MCT1 as well as MCT4 content is related to the lactate exchange ability and (ii) that MCT1 would be related to the lactate removal ability during recovery.

## Methods

### Subjects

Eighteen well-trained to highly-trained lightweight rowers volunteered to participate in the study. Their mean (± SD) age, height and weight were 22 ± 3 year, 180 ± 5 cm, 72.1 ± 3.1 kg, respectively. According to their experience, they trained between 5 and 12 times a week for 5 and 11 years. Four of them were world champions. The experiment was conducted in accordance with the Declaration of Helsinki regarding the use of human subjects and was approved by the Committee for Protection of Human Subjects of the University Hospital of Saint-Etienne, France. Before giving their written informed consent, subjects were advised of the objectives, all risks, possible discomforts and potential benefits of the experiment.

### Experimental design and equipment

The experiments comprised three visits, carried out at least 3 days apart. Subjects were instructed to not undertake any strenuous activity during the 24 h preceding the visits. All tests were performed on a rowing ergometer (Model D, Concept2, Morrisville, VT, USA), with which the rowers are fully familiar. The computer of the ergometer continuously delivered the work and stroke rates (in W and min^−1^, respectively).

#### Visit 1: incremental exercise up to exhaustion

This graded exercise started at 150 W, with power output incremented by 50 W steps until volitional exhaustion. Each step consisted of 3 min rowing and two consecutive steps were separated by 0.5 min rest. Blood samples were taken from the earlobe at rest, during the rest interval between two steps and 3 min after exercise completion (considered as peak value) to determine blood lactate concentration ([La]_b_, in mmol·L^−1^). Expired gases were sampled during the last 30 s of each step, to measure oxygen uptake ($\dot{V}$O_2_, in L·min^−1^). Heart rate (HR, in beats·min^−1^) was measured continuously. The session was intended to determine peak oxygen uptake ($\dot{V}$O_2peak_, in L·min^−1^ and mL·kg^−1^ · min^−1^), the mechanical power output corresponding to $\dot{V}$O_2peak_ (Pa_peak_, in W) and the blood lactate response to incremental exercise.

#### Visit 2: 3-min all-out exercise

Hyperemic cold cream (Dolpyc®) was applied to the earlobe, and subjects performed a 10-min warm-up at 130 beats·min^−1^ (~50% of Pa_peak_). After a 2-min rest, they performed a 3-min all-out test (no constant work rate), followed by 90 min passive recovery. Subjects remained seated during the rest and recovery periods. Blood samples were collected from the earlobe at rest ([La]_rest_, in mmol·L^−1^), end of warm-up ([La]_warm−up_, in mmol·L^−1^), exercise completion ([La](0), in mmol·L^−1^) and at 0.5, 1, 1.5, 2, 2.5, 3, 3.5, 4, 4.5, 5, 6, 8, 10, 12, 15, 20, 25, 30, 40, 50, 60, 70, 80, and 90 min of recovery. peak [La]_b_ values (in mmol·L^−1^) and the corresponding time (in min) were recorded ([La]_peak_ and *t*[La]_peak_, respectively). This session allowed determination of mean power output [expressed in absolute value (P_AO_, in W) and relative to Pa_peak_ (%P_AO_)] and individual blood lactate recovery curve. This type of exercise was chosen because it involves maximally the nonoxidative glycolytic energy pathway (Medbø et al., 1988).

#### Visit 3: muscle biopsy

Biopsy was taken at rest from the vastus lateralis (at a level corresponding to one third of the distance from the upper margin of the patella to the anterior superior iliac supine) of the right leg. After a small incision was made in the skin and fascia under local anesthesia (Xylocaïne 2%), tissues were harvested using Weil-Blakesley forceps. Muscle biopsies were divided into three parts. One part was mounted in Tissue-Tek II O.C.T. for histochemical and immunohistochemical analyses. The two other parts were immediately frozen and stored in liquid nitrogen until analysis for enzyme activities measurements and MCTs content determination by Western blots.

### Measurements and calculation

#### Cardio-ventilatory and metabolic measurements

After passing through a three-way low-resistance mouthpiece (Hans Rudolph 2700, Hans Rudolph Inc., Kansas City, MO, USA) and a low dead-space mixing chamber, the expired gases were collected in a balanced Tissot spirometer for flow measurement. The O_2_ and CO_2_ fractions were measured in ambient air and in the mixing chamber by D-Fend Datex (Helsinki, Finland) and S3A/I Ametek (Pittsburgh, PA, USA) analyzers, respectively. These analyzers were pre-calibrated on precision-analyzed gas mixtures. The expired air volumes and O_2_ and CO_2_ fractions were recorded to calculate $\dot{V}$O_2_. $\dot{V}$O_2peak_ was the highest recorded $\dot{V}$O_2_. HR was determined by electrocardiogram analysis (Cardimax FX-121 Electrocardiograph, Fukuda Denshi, Tokyo, Japan). Arterialized capillary blood was sampled by micropunctures (20 μL) at the earlobe, then diluted in a hemolysing solution and stored at 4°C until analysis. Lactate concentration was determined enzymatically in whole blood, using the YSI 2300 lactate analyzer (YSI Inc., Yellow Springs, OH, USA).

#### Parameters determination

Pa_peak_ was determined by linear interpolation from the $\dot{V}{\text{O}}_{2}$ versus power output curve.

### Bi-compartmental model

Individual blood lactate recovery curves were fitted to the bi-exponential time function (Equation 1) where: [La](0) and La(t) (mmol·L^−1^) are lactate concentrations in capillary blood measured at exercise completion and at a given recovery time, respectively; A_1_ and A_2_ (mmol·L^−1^) are the amplitudes of the exponential terms; and γ_1_ and γ_2_ (min^−1^) are the velocity constants describing the lactate exchange and removal abilities, respectively (Freund and Zouloumian, 1981a,b). Blood lactate recovery curves were fitted to Equation (1) by iterative non-linear regression technique on KaleidaGraph 4.0 software (Synergy Software, Reading, PA, USA) to determine the values of A_1_, A_2_, γ_1_, and γ_2_, [La](0) being an experimental measurement.

Recently, we (Maciejewski et al., 2013) developed a method to estimate the amount of lactate accumulated in the body at the end of exercise (QLaA, in mmol):

(2) $$\begin{array}{l} \text{QLaA}=\text{QLaA at }{\left[\text{La}\right]}_{\text{peak}}+\text{QLaR} \end{array}$$

where QLaA at [La]_peak_ is the quantity of lactate accumulated at [La]_peak_ which is calculated as the following:

(3) $$\begin{array}{l} \text{QLaA at }{\left[\text{La}\right]}_{\text{peak}}={\left[\text{La}\right]}_{\text{peak}}\cdot {\text{V}}_{\text{TLS}} \end{array}$$

where [La]_peak_ represents the maximal blood lactate concentration during recovery and V_TLS_ represents the total lactate distribution space (i.e., 600 mL · kg^−1^). QLaR represents the amount of lactate removed at the end of exercise to [La]_peak_, which is calculated as:

(4) $$\begin{array}{l} \text{QLaR}=\left[\left({\left[\text{La}\right]}_{\text{peak}}+\left[\text{La}\right]\left(0\right)\right)∕2\right]\cdot \gamma_{2}\cdot \text{t}{\left[\text{La}\right]}_{\text{peak}}\cdot {\text{V}}_{\text{TLS}} \end{array}$$

where *t*[La]_peak_ is the time to reach the maximal lactate concentration during recovery. For more information about this method, we refer the reader to Maciejewski et al. (2013).

Lactate removal rate at the end of exercise [LRR(0), in mmol^·^min^−1^] was estimated from the following equation:

(5) $$\begin{array}{l} \text{LRR}\left(0\right)=\gamma_{2}\cdot \text{QLaA} \end{array}$$

### Muscle analysis

#### Fiber types distribution

Frozen serial cross sections of 10 μm thickness were cut using a microtome at −20°C (HM 560, Microm, Walldorf, Germany). Then, two complementary methods have been used to accurately determine skeletal muscle fiber types. In the first method, fiber type was studied on serial sections stained for myofibrillar ATPase after preincubation at different pH (pH 4.35, 4.55, and 10.4), according to the methodology of Brooke and Kaiser (1970). In the second method, fiber typology was studied on immunohistochemical serial preparations using anti-fast IIa myosin heavy chain N2.261 (Alexis Biochemical) and anti-slow myosin heavy chain A4.951 (Alexis Biochemical) monoclonal antibodies. The fibers were designated as I, IIa and IIx.

#### Microvascular network

The identification of microvessels was performed using the monoclonal antibody CD31 (Dako, Glostrup, Denmark; M0823), which recognizes platelet endothelial cell adhesion molecule-1, a transmembraneous glycoprotein strongly expressed by vascular endothelial cells. The slices were incubated in an atmosphere saturated with water vapor, for 1 h with CD31 antibody (mouse anti-human), for 30 min with the secondary antibody (rabbit anti-mouse, Dako, P0260) and for 30 min with the tertiary antibody (swine anti-rabbit, Dako, P0217). All incubations were performed at room temperature and the slides were rinsed between incubation with a phosphate-buffered saline solution. Peroxidase labeling was performed using a DAB substrate kit (Vector, Burlingame, USA; SK-4100), that yields a brown reaction end-product at the site of the target antigen.

#### Microscopy and analyses

Muscle sections were viewed under a light microscope (Eclipse E400, Nikon, Badhoevedorp, Netherlands) connected to a digital camera (Coolpix 990, Nikon). Photographs were taken at different magnifications (from x40 to x400) for (i) fiber type, (ii) cytochrome c oxidase (COx) activity, and (iii) capillary density (CD). For these analyses, an average of eight fields was examined in each section, allowing for inspection of 150 fibers per individual sample. All photographs were analyzed using Scion image analysis free software (Scion Image Software, Scion Corporation, Frederick, Maryland, USA).

#### Capillary density assessment

In a given area, the number of capillaries was counted, and the CD was expressed as the number of capillaries per square millimeter (cap·mm^−2^). The mean number of capillaries around a single fiber (CAF) was also calculated.

#### Enzyme activity analysis

One portion of the muscle sample (~30 mg) was freeze-dried, dissected free of connective tissue and blood and then powdered. The muscle powder was weighed at room temperature in a glove box where the hygrometry was lower than 40%. Tissue was then homogenized at 4°C in 0.1 M phosphate buffer (pH 8.2) containing 5 mM 2-mercaptoethanol, 30 mM NaF, 5 mM MgCl_2_, and 0.5 mM ATP. An aliquot of this tissue suspension was immediately used to measure spectrophotometrically the activity of creatine kinase (CK), phosphofructokinase (PFK), lactate dehydrogenase (LDH), citrate synthase (CS) and β-hydroxyacyl-CoA-dehydrogenase (β-HAD). These enzymes activities were measured at 25°C and were expressed in μmol·min^−1^.g^−1^ of dry muscle. COx activity was determined on muscle sections. The slides were incubated for 120 min at 37°C in 0.05 M phosphate buffer (pH 7.3) containing 20 mg 3,3′–diaminobenzidine tetrahydrochloride (Sigma-Aldrich), 140 mg cytochorme *c* (Sigma Biochemical, Poole), 3 g saccharose (Carlo Erba Reactive-SDS), and 4 mL of catalase (Sigma-Aldrich) solution. After incubation, slides were rinsed three times in distilled water and dehydrated in three different alcohol baths. Measurements of COx activity were performed by converting the image to gray scale to determine optical density using an image analysis free software (Scion Image Software, Scion Corporation, Frederick, Maryland, USA). The mean relative optical density per pixel was determined by subtracting the background. The fiber optical density was proportional to its oxidative capacity: the darker the fiber, the higher the COx activity. For each subject, a single value per structure was obtained by averaging measurements from a mean of 150 well-identified muscular fibers.

#### Western blotting for MCT1 and MCT4 proteins quantification

The last proportion of muscle (> 30 mg) was homogenized (Polytron 2100, Kinematica, Inc., Newark, NJ, USA) at 4°C in a buffer A (210 mM sucrose, 2 mM EGTA, 40 mM NaCl, and 30 mM HEPES, pH 7.4) supplemented with 0.15% protease inhibitor cocktail (Sigma P-8340). The homogenate was centrifuged at 600 *g* for 10 min at 4°C to remove heavy material, including a fraction of the mitochondria. The supernatant was diluted with 0.75 vol. of a buffer B (1.167 M KCl and 58.3 mM Na_4_P_2_O_7_.10H_2_O, pH 7.4) and centrifuged at 230,000 *g* for 60 min at 4°C. The new supernatant (cytosolic fraction) was stored at −80°C, while the resulting pellet (total membrane fraction including sarcolemmal and mitochondrial membrane fractions) was resuspended in a buffer C (1 mM EDTA and 10 mM Tris, pH 7.4). The suspension was then mixed with 0.33 vol. of 16% SDS and centrifuged at 1100 *g* for 25 min at room temperature to eliminate insoluble materials. Proteins content was then determined using bovine serum albumin (BSA) as a standard (Pierce). Supernatants were divided into aliquots and stored at −78°C for protein assays and immunoblotting. Sample of muscle homogenates (15 μg of protein) and molecular weight marker (Bio-Rad) were separated on a 10% SDS-PAGE and electroblotted onto polyvinylidene difluoride (PVDF) membranes. The membranes were blocked overnight at 4°C in a buffer D (150 mM NaCl, 10% nonfat dried milk, 0.1% Tween 20 and 50 mM Tris, pH 7.5) and then incubated with either antibodies against MCT1 (0.1 μg.mL^−1^) or MCT4 (0.2 μg.mL^−1^) for 2 h at room temperature in buffer D. The antibodies were gifts from Professor J. Mercier (University of Montpellier 1, France) home-made as previously described (Bishop et al., 2007; Thomas et al., 2007). After a 15-min wash followed by two 5-min washes in buffer E (150 mM NaCl, 0.1% Tween 20 and 50 mM Tris, pH 7.5), membranes were incubated for 60 min at room temperature with anti-rabbit secondary antibody in buffer E. Membranes were then washed as described above, and MCT1 or MCT4 expression was detected by enhanced chemiluminescence (ECL) detection according to the manufacturer's instruction (Renaissance, NEN). Films were scanned (VXR-12 Plus Film Digitizer, VIDAR System Corporation, Herndon, USA), and band intensities were quantified with Scion Image software (Scion Image Software, Scion Corporation, USA). Individual muscle MCT1 and MCT4 contents were expressed in percentage of a same standard sample loaded on each gel. For each subject, a single value of MCT1 and MCT4 expression was obtained by averaging measurements from 4 different Western blots.

### Statistical analysis

Analyses were performed using JMP V9.0.1 (SAS Institute, Cary, NC, USA). Descriptive statistics are expressed as means ± standard deviation (SD). Relationships between two variables were studied by means of linear regressions (confirmed by Pearson tests). Analysis by multiple regressions has also been used. Correlations among variables were considered to be significant for *P* < 0.05.

## Results

### Incremental exercise up to exhaustion

Exhaustion intervene after 16.8 ± 1.8 min. $\dot{V}$O_2peak_ and Pa_peak_ were 4.8 ± 0.4 L·min^−1^ (66.5 ± 3.9 mL·kg·min^−1^) and 347 ± 37 W. Mean heart rate at exhaustion and peak lactate concentration were 193 ± 8 min^−1^ and 13.4 ± 2.4 mmol·L^−1^, respectively. $\dot{V}$O_2_ and work rate at 4 mmol·L^−1^ of blood lactate concentration were 4.2 ± 0.5 L·min^−1^ 284 ± 35 W, respectively. These latter values corresponded to 88.1 ± 5.2% and 81.7 ± 4.4% of $\dot{V}$O_2peak_ and Pa_peak_, respectively.

### 3-min all-out exercise

The mean absolute power output (P_AO_) was 424 ± 26 W (123 ± 8% of Pa_peak_) The mean values of [La]_rest_ and [La]_warm−up_ were 1.3 ± 0.3 and 1.2 ± 0.2 mmol·L^−1^, respectively. The mean value of [La]_b_ at the end of exercise ([La](0)) is reported in Table 1. The peak [La]_b_ values ([La]_peak_) and the time to reach [La]_peak_ (*t*[La]_peak_) were 18.0 ± 2.6 mmol·L^−1^ and 7.5 ± 1.9 min, respectively.

**Table 1:** **Mean values of the blood lactate kinetics parameters obtained during the recovery period after a 3-min all-out exercise**.

	**[La](0) (mmol·L^−1^)**	**A_1_ (mmol·L^−1^)**	**γ_1_ (min^−1^)**	**A_2_ (mmol·L^−1^)**	**γ_2_ (min^−1^)**
Mean ± SD	12.3 ± 2.4	17.5 ± 6. 5	0.179 ± 0.049	−29.3 ± 8.3	0.0336 ± 0.0077

*La(0) is the blood lactate concentration at end of exercise; A_1_ and A_2_ are the amplitudes of concentration variations; γ_1_ and γ_2_ are the velocity constants*.

Equation (1) accounted for more than 99% of the variance of the experimental blood lactate recovery curves. The mean values of the blood lactate curve parameters A_1_, A_2_, γ_1_, and γ_2_ are reported in Table 1. Mean QLaA and LRR(0) were 945 ± 153 mmol and 31. 7 ± 8.7 mmol·min^−1^, respectively.

### Skeletal muscle characteristics

**Figure 1:**
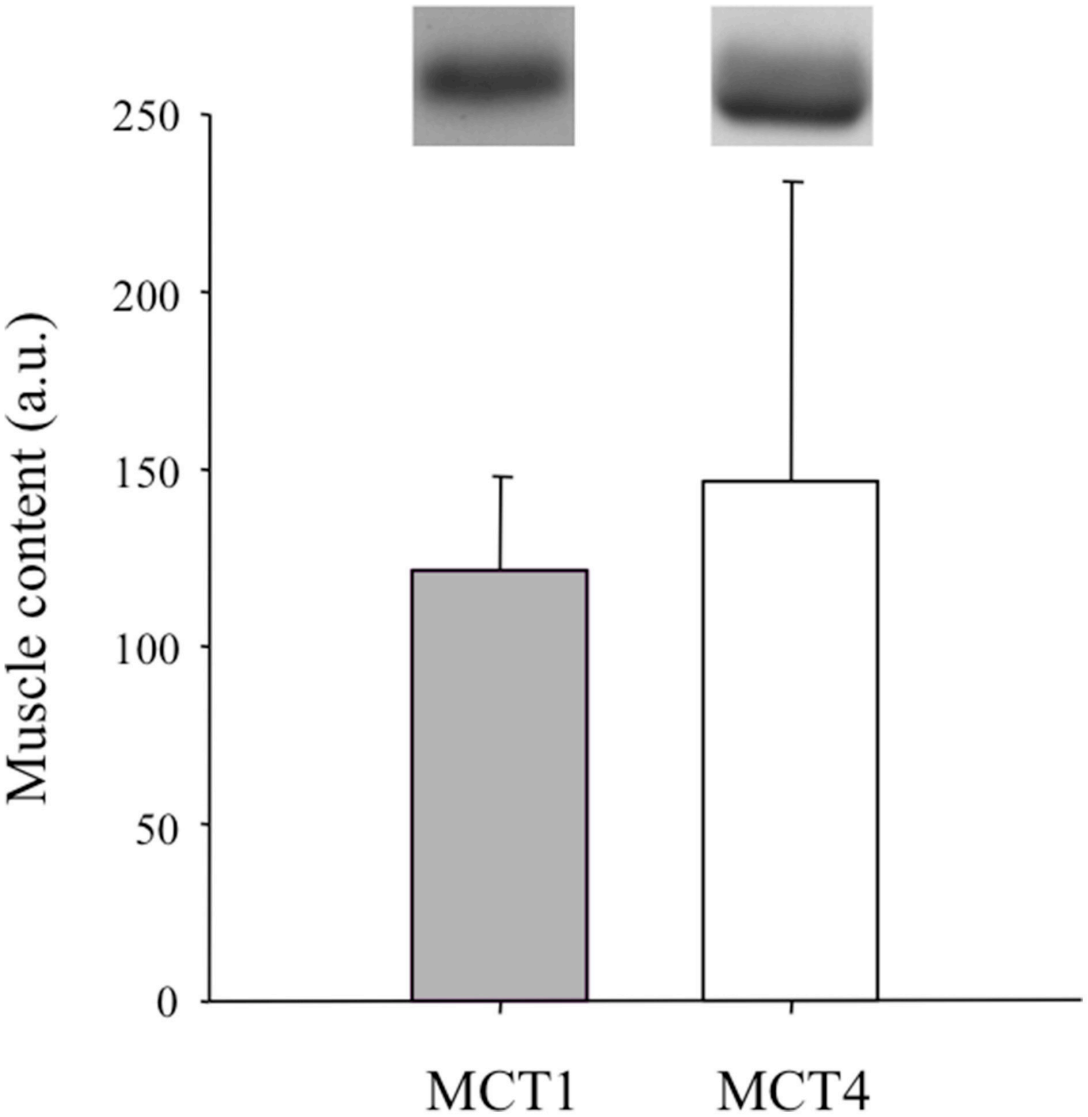
**Muscle MCT1 and MCT4 content (a.u.: arbitrary units)**.

Muscle fiber types distribution was 68.6 ± 10.8%, 31.4 ± 10.8%, and 0.0 ± 0.2% for types I, IIa, and IIx, respectively. The activities of some skeletal muscle key enzymes are reported in Table 2. CD was on average of 398 ± 42 capillaries per mm^2^. The mean value of CAF was 6.11 ± 1.18 capillaries. Muscle MCT1 and MCT4 content are reported in Figure 1.

**Table 2:** **Metabolic enzymes activities in vastus lateralis muscle of highly trained lightweight rowers**.

	**CK**	**PFK**	**LDH**	**CS**	**β-HAD**	**COx**	
						**I**	**IIa**
	**(μmol·min^−1^ · g dry muscle^−1^)**					**(a.u.)**	
Mean ± SD	1229.63 ± 91.58	144.09 ± 34.28	431.38 ± 97.94	76.31 ± 15.01	22.65 ± 2.41	27.74 ± 3.80	22.93 ± 4.63

*CK, PKF, and LDH are creatine kinase, phosphofructokinase and lactate dehydrogenase, respectively; CS and β-HAD are citrate synthase and β-hydroxyacyl-CoA-dehydrogenase, respectively. COx is cytochrome-c oxidase activity (a.u.: arbitrary unit; optical density)*.

### Correlations among variables

**Figure 2:**
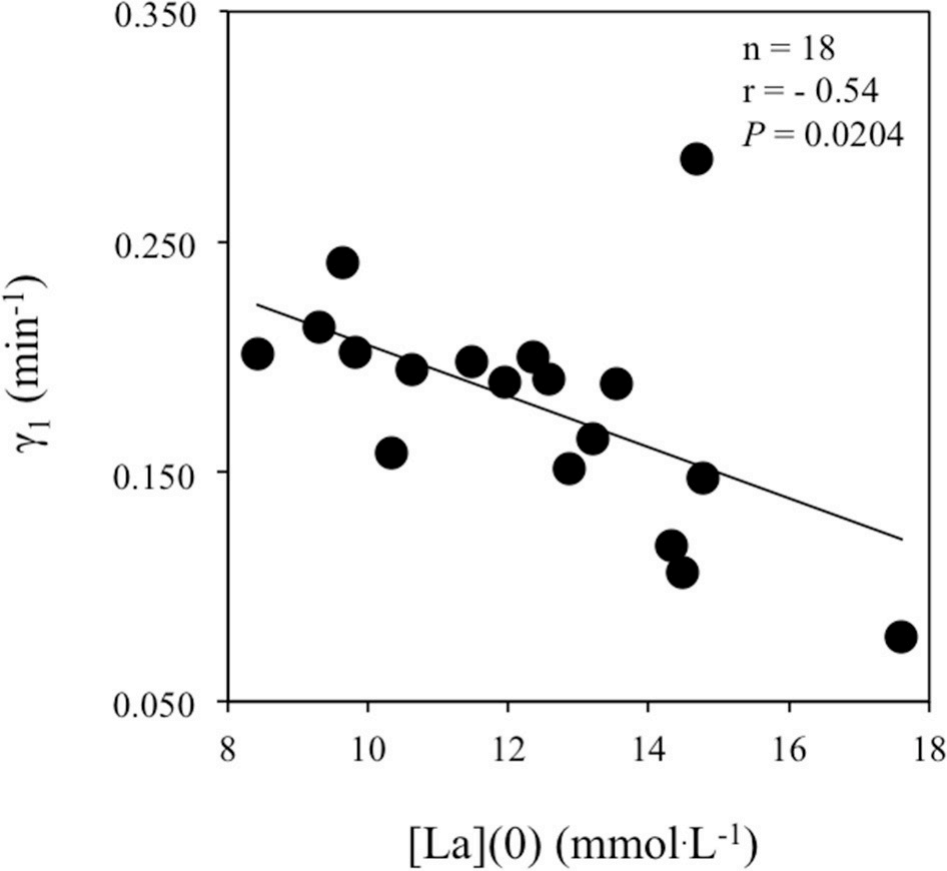
**Correlation between the velocity constant γ_1_ (min^−1^) and the blood lactate concentration at exercise completion i.e., [La](0) (mmol.L^−1^)**.

**Figure 3:**
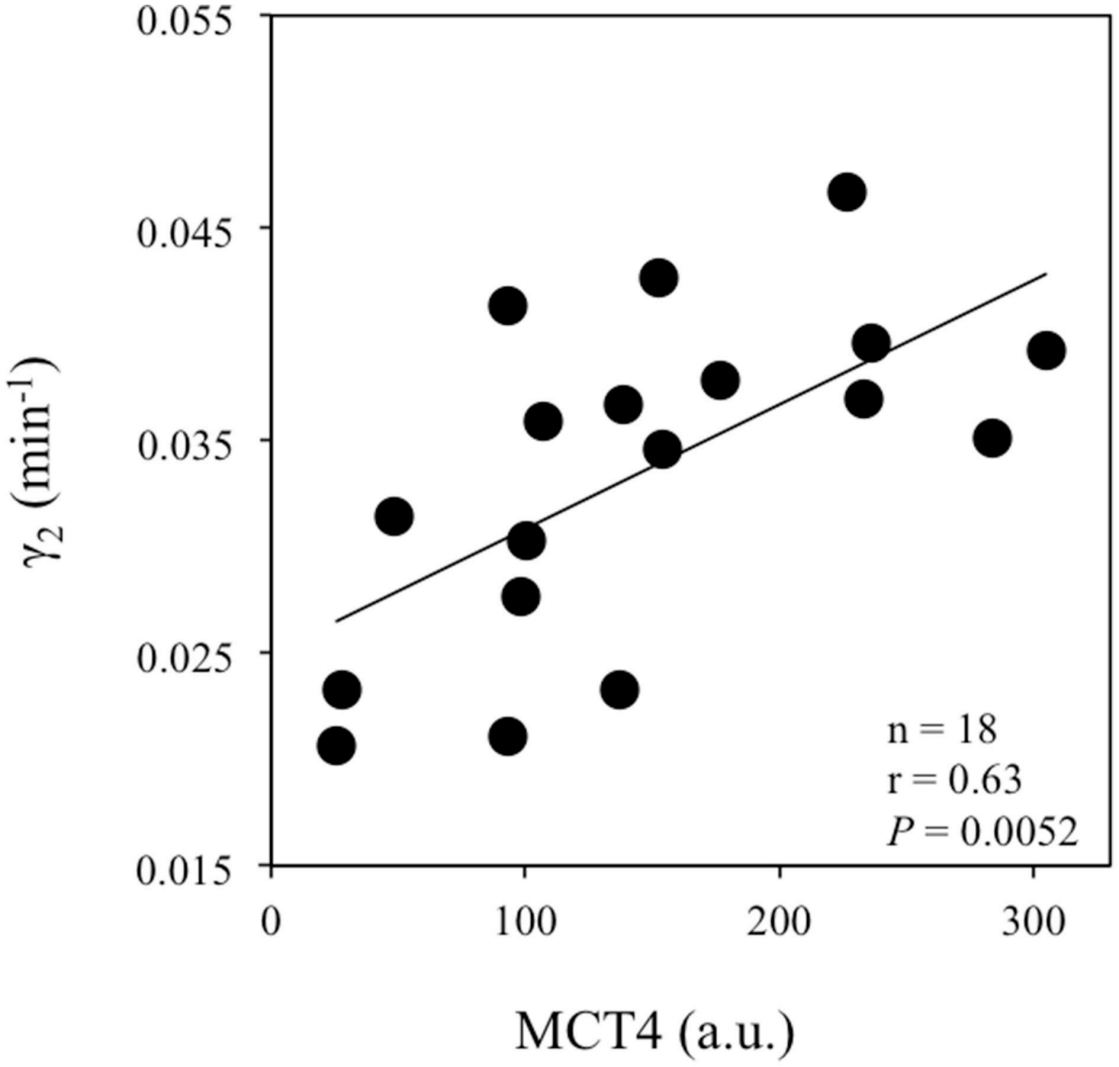
**Correlation between the velocity constant γ_2_ (min^−1^) and the *vastus lateralis* muscle MCT4 content (a.u.: arbitrary units)**.

**Figure 4:**
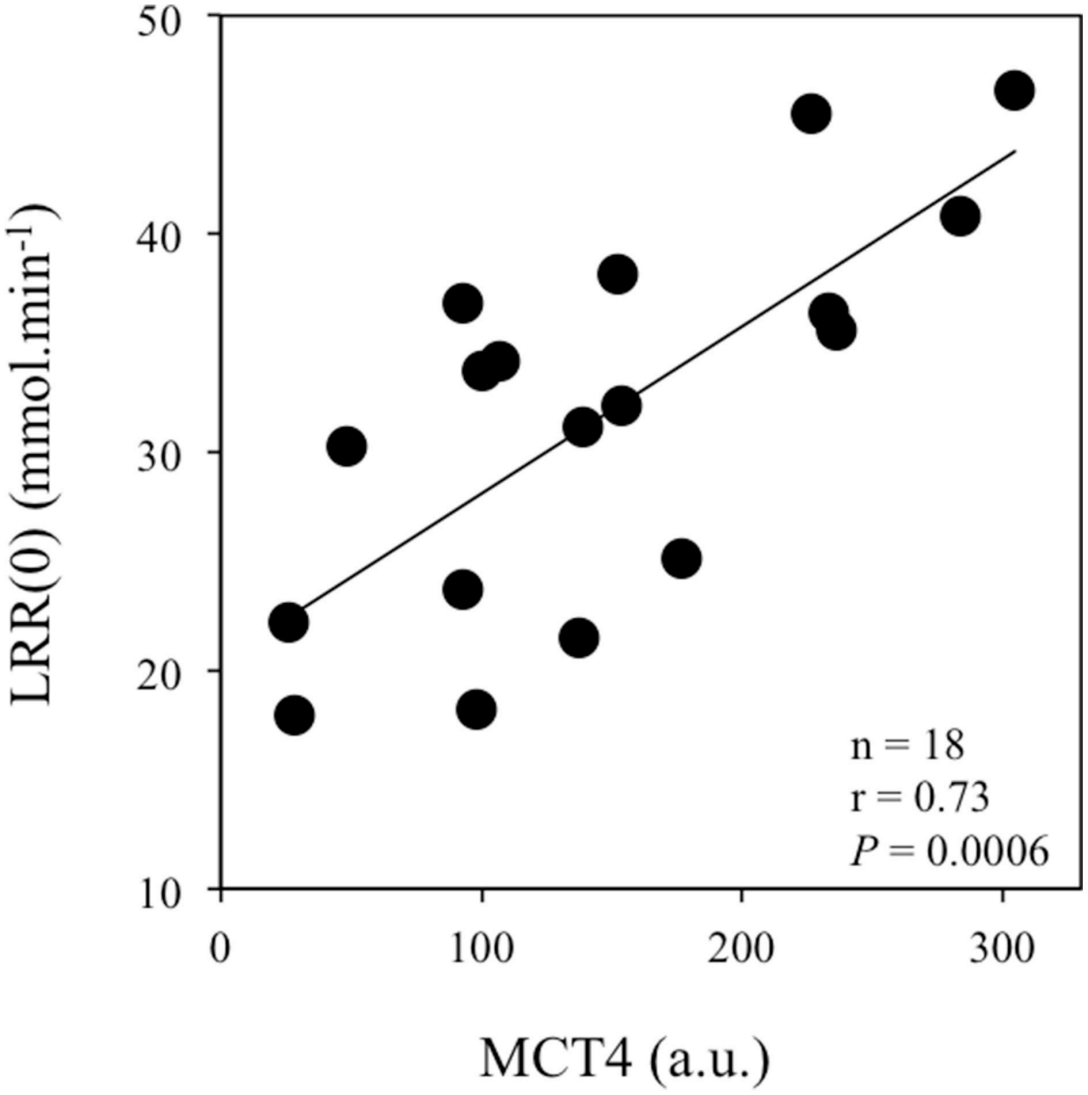
**Correlation between the lactate disappearance rate at exercise completion [LRR(0), in mmol·min^−1^] and the *vastus lateralis* muscle MCT4 content (a.u.: arbitrary units)**.

Table 3 reports correlations between the lactate kinetic [γ_1_, γ_2_, La(0) and LRR(0)] and transport (muscle MCT1 and MCT4 content) parameters. The velocity constant γ_1_ was correlated with La(0) (Figure 2) but not with MCT1, MCT4 or CD. The velocity constant γ_2_ (Figure 3) and LRR(0) (Figure 4) were correlated with the muscle content of MCT4 but not with that of MCT1. No correlations between muscle characteristics and lactate kinetic or transport parameters were noticed.

**Table 3:** **Correlation matrix of the blood lactate recovery kinetic parameters and the muscle lactate/H^+^ co-transporters content**.

	**γ_1_ (min^−1^)**	**γ_2_ (min^−1^)**	**LRR(0) (mmol·min^−1^)**	**MCT1 (a.u.)**	**MCT4 (a.u.)**
[La](0)	*r* = −0.54	*r* = 0.03	*r* = 0.42	*r* = 0.38	*r* = 0.43
(mmol·L^−1^)	*P* < 0.05	*NS*	*P < 0.1*	*NS*	*P* < 0.1
γ_1_	–	*r* = 0.02	*r* = 0.31	*r* = 0.01	*r* = 0.28
(min^−1^)	–	*NS*	*NS*	*NS*	*NS*
γ_2_	–	–	*r* = 0.82	*r* = 0.13	*r* = 0.63
(min^−1^)	–	–	*P* < 0.001	*NS*	*P* < 0.01
LRR(0)	–	–	–	*r* = 0.03	*r* = 0.73
(mmol·min^−1^)	–	–	–	*NS*	*P* < 0.001
MCT1	–	–	–	–	*r* = 0.30
(a.u.)	–	–	–	–	*NS*

*La(0) is the blood lactate concentration at end of exercise; γ_1_ and γ_2_ are the velocity constants; LRR(0) is the lactate removal rate at the end of exercise; MCT1 and MCT4 are lactate/H^+^ co-transporters (a.u.: arbitrary unit)*.

## Discussion

The first important finding of the present study was that, contrary to our hypothesis, muscle MCT1 and MCT4 content was not correlated with γ_1_ i.e., the lactate exchange ability. The second important finding of the present study was that the muscle MCT4 content of our trained rowers was positively correlated with γ_2_ i.e., the lactate removal ability during recovery.

### Muscle MCTs content and lactate exchange ability

In the present study, we investigated the possible relationship between the lactate exchange ability from the previously active muscles and the blood during the first minutes of recovery and the muscle MCTs content. Contrary to our hypothesis, no correlation was observed between γ_1_ and the muscle MCT1 and/or MCT4 content. In the past, previous studies have already reported that muscle MCTs content may dissociate from the lactate transport capacity (Tonouchi et al., 2002; Lambert et al., 2003), which efficiency has a physiological meaning close to γ_1_ (Juel, 1997). Furthermore, it is important to keep in mind that γ_1_ is an integrative parameter determined *in vivo* which depends besides to the muscle MCTs content, on the functionality of the carriers which is driven by the milieu, additional (i.e., other than MCTs-related) pH regulation mechanisms, capillary density and local blood flow (Tesch and Wright, 1983; Pilegaard et al., 1995; Juel, 1997; Nielsen et al., 2002a; Messonnier et al., 2007).

The γ_1_ values obtained in the present study after maximal exercise are the lowest ever reported in the literature while the highest γ_1_ values ever reported after 90% $\dot{V}$O_2peak_ exercises have been obtained in trained rowers (Messonnier et al., 1997). This suggests that the lactate exchange ability was deeply altered in the present study by the previously performed (i.e., all-out supramaximal) exercise. Interestingly, we observed an inverse correlation between γ_1_ and [La](0) (Figure 2). Because it is unlikely that this correlation means that the higher the lactate exchange ability the lower the blood lactate concentration at exercise completion, an alternate explanation would be that lactate levels and/or accompanying changes in pH may alter lactate transport. In accordance with this interpretation, Juel (1997) has reported that for a same muscle-to-blood lactate gradient, the net lactate efflux from giant sarcolemmal vesicles decreased when lactate concentrations increased, suggesting a deleterious role of the lactate concentrations *per se* on the efficiency of the transporters. By extension, the correlation (Figure 2) suggests that the functionality of the carriers seems to be more important than their muscle content to determine lactate transport after maximal all-out exercise. Consistent with this latter inference, Nielsen et al. (2002a) who used bicarbonate to induce blood alkalosis i.e., a positive alteration of milieu driving lactate transport via changes in muscle-to-blood pH gradient, observed striking increases in muscle lactate release and blood lactate accumulation during maximal rowing exercise.

One cannot exclude the fact that the large muscle mass involved in rowing contributes to the profound alteration of γ_1_ observed in the present study. Diverging from classically studied activities, i.e., cycling or running, rowing involves typically 70–80% of body muscle mass (Steinacker, 1993), that alters substantially the diffusion space for lactate and H^+^ released from the active muscles. Consequently, the diffusion space is rapidly loaded by these ions in rowing that may, according to the relationship between γ_1_ and the milieu (Figure 2), explained the low γ_1_ values reported here.

### Muscle MCTs content and lactate removal ability during recovery

Previously, it has been shown that the velocity constant γ_2_, which denotes the lactate removal ability during recovery, was significantly correlated with the citrate synthase activity, mitochondrial respiration and muscle content of MCT1 (Thomas et al., 2005). These as already mentioned in the introduction, previous studies (Thomas et al., 2005) supported the idea (i) that oxidation may be a major pathway for lactate removal during recovery (Brooks et al., 1973; Brooks, 2009) and that MCT1 is involved in the uptake of lactate by consumer tissues including the previously active muscles (Juel and Halestrap, 1999; Bonen et al., 2000), and (ii) that genes of MCT1 and markers of muscle oxidative capacity share a common transcriptional regulatory mechanism, probably via PGC-1α (Benton et al., 2008). In the present study, no correlation between the velocity constant γ_2_ and the Cox activity (Hashimoto et al., 2006) or the muscle content of MCT1 (Thomas et al., 2005) was observed. This lack of correlations may lie on (i) the important muscle mass involved in rowing, so that COx activity and MCT1 content of the vastus lateralis might not be representative of the whole muscle mass involved in exercise, (ii) an important glycogen repletion in the previously active muscles, and (iii) gluconeogenesis or oxidation in other tissues (e.g., liver and heart).

An unexpected result of the present study was the positive correlation between γ_2_ and the muscle content of MCT4 (Figure 3). Interestingly, LRR(0) was also correlated with muscle MCT4 content (Figure 4). Taken together, these results suggest that MCT4 may at some point constitute a potential rate-limiting step in the lactate removal processes during recovery following extremely high-intensity exercise. Literature has provided arguments in favor of the fact that MCT4 would be mainly involved in the extrusion of lactate and H^+^ from the muscle cell rather than in their uptake (Dimmer et al., 2000; Halestrap, 2013). However, it is important to keep in mind that MCT4 is a bidirectional lactate/proton co-transporter with a much higher affinity for lactate than for pyruvate [*K*_*m*_ ~30 mM for lactate vs. ~150 mM for pyruvate (Dimmer et al., 2000; Halestrap, 2013)]. Therefore, during recovery after supramaximal rowing exercise, when extracellular lactate concentrations are extremely elevated (Nielsen, 1999) as it is the case in the present study and approximately 30-fold higher than those of pyruvate (Baron et al., 2008), it is conceivable that once the net lactate release rate is almost nil [i.e., after ~10 min into recovery (Freund et al., 1984; Juel et al., 1990; Bangsbo et al., 1991, 1993)], MCT4 may also facilitate lactate entry into consumer tissues including myocytes for subsequent utilization. In accordance with this framework, previous experiments clearly indicated that lactate uptake by consumer tissues and disappearance are directly related to lactate delivery and blood concentrations (Miller et al., 2002; Nielsen et al., 2002b; Van Hall et al., 2003; Messonnier et al., 2013). As a whole, the extremely hard exercise performed in the present study and the high lactate accumulation at exercise completion may reach levels that are compatible with a possible role of MCT4 in the influx of lactate into tissues for subsequent removal. One might also consider the correlation between γ_2_ and MCT4 as a more indirect link. Following this line of reasoning, the muscle content of MCT4, which delineates the first step (i.e., the extrusion of lactate from the lactate-loaded myocytes) in the distribution of lactate toward other cells/tissues, could be a potential rate-limiting step for subsequent removal, which is elevated in trained athletes (Messonnier et al., 1997, 2013). Although the present study does not allow to determine precisely the mechanisms, our results suggest nevertheless that after all-out high-intensity exercises leading to extreme lactate accumulation, MCT4 may play a significant role in lactate transport/distribution/delivery for subsequent removal.

### Particular population

The possible role of MCT4 drawn by the present study in the fate of lactate during recovery might also be related to the studied population, i.e., well-trained rowers. Indeed, our knowledge of the effects of long-term training on the functional role of MCT1 and MCT4 is relatively incomplete (Thomas et al., 2011). If muscle MCT1 and MCT4 content increases in response to training (Dubouchaud et al., 2000; Evertsen et al., 2001), available data until recently suggested that this improvement might be limited to the first 6–8 weeks of intense training and that years of training might have no further or at least limited effects (Juel, 2008). However, a recent investigation (Neal et al., 2013) demonstrated that polarized (80% low intensity, 0% moderate intensity, 20% high intensity) and threshold (55% low intensity, 45% moderate intensity, 0% high intensity) training programs over 6 weeks improved muscle MCT4 but not MCT1 content in well-trained cyclists. If MCT4 is subjected to training-induced adaptations when MCT1 content does not respond anymore, one can not exclude that the long-term training history of the present rowers might have induced specific adaptations explaining, at least in part, an influence of muscle MCT4 content on the lactate removal processes (i.e., γ_2_) during recovery following an all-out exercise leading to maximal tolerable lactate accumulation. In that sense, it is worthy to note that the experienced rowers involved in the present study were endurance-trained for more than 4 years and characterized by elevated muscle oxidative capacities as illustrated by their (i) high values of $\dot{V}$O_2peak_ (66.5 ± 3.9 mL·kg^−1^ · min^−1^), (ii) elevated oxidative enzymes activity (e.g., CS activity, Table 2), (iii) highly rarefied type IIx muscle fibers [that is not surprising in elite rowers (Larsson and Forsberg, 1980)], and (iv) dense capillary network (6.11 ± 1.18 capillaries per fiber). At that time, it is important to keep in mind that the results of the present study and their interpretation are totally concordant with those of previous studies showing the importance of the lactate removal processes (Messonnier et al., 1997, 2013) and muscle MCT4 content in performance achievement (Messonnier et al., 2007). Furthermore, the positive relationship reported in sub-elite athletes between performance over a 10 km running trial and muscle MCT4 content (Harley et al., 2009) reinforces the need to reappraise the role of MCT4 in lactate kinetics during events performed by highly trained endurance athletes.

### Unsolved question

One question to resolve in a near future is to understand why there is a so large dispersion in muscle MCT4 content among subjects (Pilegaard et al., 1999; Dubouchaud et al., 2000) including within a homogeneous group of trained athletes [(Harley et al., 2009; Neal et al., 2013) and present study]. Indeed, it remains surprising (Harley et al., 2009; Neal et al., 2013) and present study] that subjects with similar training history and actual schedule display such divergent muscle MCT4 contents (Figure 1).

## Conclusions

The first important finding of the present study was the lack of correlation between γ_1_ and the muscle content of MCT1 and MCT4. Taken together, the present results and those of previous experiments suggest that rather than the muscle MCTs content, the milieu, which drives the functionality of the carriers, seems to have the most important role in determination of lactate exchanges following all-out exercise. The second important finding was the correlation we observed between the velocity constant γ_2_ and the muscle content of MCT4. For the first time, MCT4 is suggested to be associated with lactate distribution and delivery for subsequent removal processes during the recovery following extremely hard exercise. Collectively, our findings and those of previous studies (Harley et al., 2009; Neal et al., 2013) suggest new insights into the role of MCT4 in lactate kinetics in well-trained athletes. However, further investigations are required to precise its functional role.

## Author contributions

HM and LM: conception and design of research; HM, MB, LF, HD, CD, HD, and LM: performed experiments; HM, MB, LF, HD, CD, and LM: analyzed data; HM, MB, LF, HD, CD, HF, and LM: interpreted results of experiments; HM and LM: prepared figures; HM and LM: drafted manuscript; HM, MB, LF, HD, CD, HF, and LM: edited and revised manuscript; HM, MB, LF, HD, CD, HF, and LM: approved final version of manuscript.

## Funding

This study was supported by a grant from the French Ministry of Youth and Sports.

### Conflict of interest statement

The authors declare that the research was conducted in the absence of any commercial or financial relationships that could be construed as a potential conflict of interest. The experiments comply with the current laws of the country in which the experiments were performed.
